# Anonymity Assurance Using Efficient Pseudonym Consumption in Internet of Vehicles

**DOI:** 10.3390/s23115217

**Published:** 2023-05-31

**Authors:** Mehreen Mushtaq, Ata Ullah, Humaira Ashraf, N.Z Jhanjhi, Mehedi Masud, Abdulmajeed Alqhatani, Mrim M. Alnfiai

**Affiliations:** 1 Department of Computer Science, National University of Modern Languages (NUML), Islamabad 44000, Pakistan; 2 Department of Computer Science and Software Engineering, International Islamic University Islamabad, Islamabad 44000, Pakistan; 3 School of Computer Science SCS, Taylor’s University, Subang Jaya 47500, Malaysia; 4 Department of Computer Science, College of Computers and Information Technology, Taif University, Taif 21944, Saudi Arabia; 5 Department of Information Systems, College of Computer Science & Information Systems, Najran University, Najran 61441, Saudi Arabia; 6 Department of Information Technology, College of Computers and Information Technology, Taif University, Taif 21944, Saudi Arabia

**Keywords:** vehicle anonymization, IoVs, pseudonym consumption, adversary, BSM, traceability

## Abstract

The Internet of vehicles (IoVs) is an innovative paradigm which ensures a safe journey by communicating with other vehicles. It involves a basic safety message (BSM) that contains sensitive information in a plain text that can be subverted by an adversary. To reduce such attacks, a pool of pseudonyms is allotted which are changed regularly in different zones or contexts. In base schemes, the BSM is sent to neighbors just by considering their speed. However, this parameter is not enough because network topology is very dynamic and vehicles can change their route at any time. This problem increases pseudonym consumption which ultimately increases communication overhead, increases traceability and has high BSM loss. This paper presents an efficient pseudonym consumption protocol (EPCP) which considers the vehicles in the same direction, and similar estimated location. The BSM is shared only to these relevant vehicles. The performance of the purposed scheme in contrast to base schemes is validated via extensive simulations. The results prove that the proposed EPCP technique outperformed compared to its counterparts in terms of pseudonym consumption, BSM loss rate and achieved traceability.

## 1. Introduction

**Figure 1:**
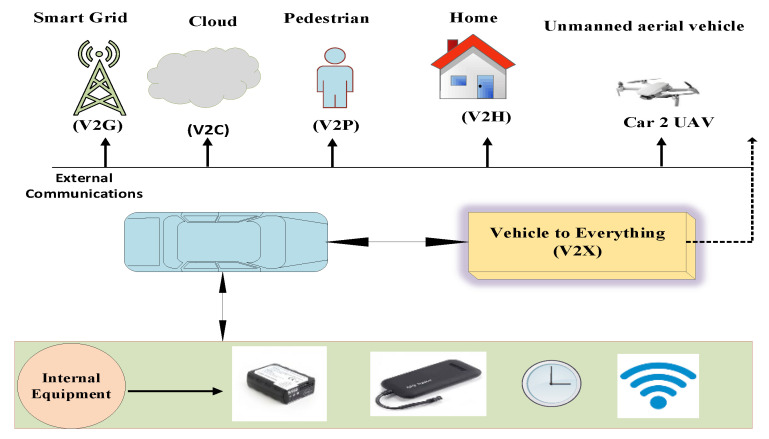
V2X communication.

Vehicular ad hoc networks (VANETs) support communication among vehicles to ensure road safety and transportation facilities by using the intelligent transport system (ITS) along with the support of road side units (RSUs) [1]. VANETs are transformed into the Internet of vehicles (IoVs) to provide more flexibility and ease to mankind. The IoVs transportation system is increasing rapidly; it is estimated that 2 billion vehicles will be connected to the IoVs by 2035. The IoVs supports five types of communication including vehicle-to-vehicle (V2V), vehicle-to-RSU (V2R), vehicle-to-infrastructure (V2I), vehicle-to-cloud (V2C) and vehicle-to-pedestrian (V2P). This communication is collectively known as vehicle to everything (V2X) communication [2,3]. The V2X communication is shown in Figure 1. The IoVs provide a set of supporting information for the drivers such as precrash warning, post-crash notification, pedestrian vicinity alert, danger zone alert and amber warning. Because of these timely notifications, the accident ratio is reduced to a large extent [4,5,6]. Besides these notifications, it provides comfort and entertainment services to both passengers and drivers [4,7].

A basic safety message (BSM) or a beacon is utilized for communication in the network. These BSMs contain all of the important information related to the vehicle (speed, velocity and direction) in plain form [8]. When this BSM is broadcasted, there is a high probability that any adversary can access this BSM. The adversary can be local or global. A local adversary is one that is part of a network, becomes a malicious node and sends network information to any other body. A global adversary is a person who eavesdrops on BSMs from their area of interest by using antennas or other devices [9]. This raises security issues and disturbs the privacy and anonymization of vehicles. An adversary can use this BSM information for bad intentions such as harming users or drivers, blackmailing or threatening them. These issues can cause hesitation in users or drivers and put their lives in danger [10].

Vehicle’s anonymity and data privacy are critical factors that cannot be compromised. To avoid these issues, a trusted authority (TA) provides pseudonyms for vehicles. Vehicles use these pseudonyms to communicate with other vehicles or RSUs [11]. These pseudonyms are changed after some time so that if an adversary is tracking a vehicle, they cannot continually trace the target vehicle’s whole trajectory. This provides security to some extent, but high pseudonym consumption makes pseudonyms insufficient. In this case, vehicles communicate to the TA directly or indirectly to issue a new set of pseudonyms [12]. This increases pseudonym consumption and computation overhead because only the TA keeps the link between the vehicle’s true identity and pseudonym [13]. It also increases the BSM loss rate, and if any safety message is lost, it results in severe consequences. So, it is important to use pseudonyms economically.

This paper presents the efficient pseudonym consumption protocol (EPCP) to use pseudonyms effectively while maintaining vehicle anonymization. In this scheme, neighbor vehicles that exist in a close range, and have the same estimated new location, are considered to be relevant vehicles. A pseudonym-changing alert is broadcasted in an efficient way after calculating the required matrices. The main contributions of our work are as follows:

(1) We explore the literature on pseudonym-based anonymity assurance for messaging in the IoVs.
(2) Next, we propose a solution to estimate the next state of vehicles and their speed and direction before sending the pseudonym-changing alert.
(3) We also deal with the exchange of pseudonyms to reduce costs and ensure anonymity as well.
(4) Finally, simulations are performed to validate the results where the proposed scheme outperforms in contrast to three dominating schemes.

The remaining part of the manuscript is organized as follows: In Section 2, the literature is discussed on pseudonym-based schemes. Section 3 provides a system model and problem statement. Section 4 presents a proposed solution. Section 5 explores the performance of the EPCP. At the end, the conclusion and future work are discussed in Section 6.

## 2. Literature Review

Many pseudonym-based schemes are presented to enhance vehicle anonymization and provide protection against attacks planned by an adversary. These techniques are majorly divided into two main classes, mix-context-based schemes and mix-zone-based schemes. In this section, schemes of both categories are discussed.

### 2.1. Mix-Context-Based Schemes

In mix-context-based schemes, vehicles change their pseudonyms together in case specified triggers are satisfied. If such triggers are not fulfilled, vehicles will not change their pseudonyms. These schemes are also known as user-centric schemes.

In [14], Pan et al. proposed a cooperative pseudonym change based on the number of neighbors (CPN) protocol. The idea behind this scheme is that vehicles tend to change pseudonyms after getting triggered. This technique increases anonymity during dense traffic flow; however, it has high pseudonym consumption. Babaghyou et al. proposed a strategy [15] in which the transmission range of vehicles was restricted as per the speed of the neighboring vehicle. The advantage of this scheme is that safety-oriented messages are not neglected. The drawback of this scheme is that pseudonym consumption is high. Vehicles that change lanes also receive BSMs, which lessens the security.

To solve the problem of pseudonym-linking, Xinghua et al. presented a scheme in which vehicles exchange pseudonyms with each other. To exchange its pseudonym, the vehicle broadcasts the request message *Req_i_* and transmits its virtual identity (VID) to the RSU. In case a nearby vehicle receives this, *Req_i_* transmits an assist reply beacon containing all of the information to the RSU [16]. This technique increases the delinking ability among the most recent and former pseudonyms, which reduces the chance of tractability. The shortcomings of the technique include high communication and computation overhead.

To reduce packet loss and reduce adversary linking attacks, Zidani et al. [17] presented a scheme in which vehicles change pseudonyms in case there is a variation in speed and on the basis of surrounding vehicles. The most prominent achievement of this scheme is that it makes use of adaptive beaconing. When the beaconing interval varies, it creates high confusion for the adversary because the adversary cannot identify when vehicles communicate and share information. The benefit of this scheme is that the adversary cannot link correctly to the pseudonyms of target vehicles.

To enhance vehicle confidentiality, cooperative pseudonym exchange and scheme permutation (CPESP) [18] is presented. This technique is a mixture of two separate schemes consisting of cooperative pseudonym exchange (CPE) and scheme permutation (SP). In the first phase, the vehicles which are ready to swap their pseudonym may broadcast a BSM to neighbor vehicles for showing willingness. In scheme permutation, vehicles change their pseudonym using two methods, which are either RSP or the periodical pseudonym-changing procedure. One technique is selected for the time being. The SP technique is considered as being highly valuable in low road traffic. In this scheme, both CPE and SP algorithms work equally. The unutilized set of pseudonyms is used in a hybrid way where one technique is chosen as the RSP, and the periodical pseudonym is considered on behalf of the pseudonym-updating process. This technique has higher protection against linking attacks, and more schemes need to be added for increasing confusion for an adversary. In [19], the technique uses three types of pseudonyms including real, initial and new pseudonyms produced by the TA, RSU and onboard unit (OBU), correspondingly. Each pseudonym is allocated to vehicles before the authentication of the previous one. The advantage of this scheme is that a pseudonym-linking attack is not possible because a pseudonym is updated by three entities, but it increases computation overhead and has very high pseudonym consumption.

To enhance privacy and maintain low traceability, the context-adaptive privacy scheme (CADS) was proposed [20]. Vehicles switch to silence while changing pseudonym; however, this silent mode is smaller to prevent missing important safety-oriented messages. The benefit of this technique is that it much lessens adversary traceability. Another technique, dynamic grouping and virtual pseudonym-changing (DGVP), was recommended to increase anonymization. The idea behind this technique is that vehicles are clustered into groups and any one of them is chosen as the group leader (GL). Each group member is allotted a group identity (GID). When vehicles are higher than a threshold value, vehicles update their pseudonym, or else a virtual pseudonym-updating mechanism is introduced [21]. The benefit of this technique is that external vehicles cannot listen to information from other group members. The problem is that the computation cost rises during the virtual pseudonym exchange due to an extra beacon being created in it.

To reduce the traceability problem, another scheme named crowd-based mix context (CMC) was proposed, in which vehicles with heavy traffic broadcast beacon messages with PU = 1 notify other vehicles to change pseudonyms. When traffic is lower, two pseudonyms are generated and exchanged randomly with each other. The neighbors accept the correct pseudonym and the false one is excluded [22]. The benefit of this technique is that the adversary cannot trace the target vehicle for a long time successfully. The drawback of the technique is that it is applicable only to vehicles moving at low speeds.

In [23], vehicles tend to change pseudonyms in groups, and these groups are monitored by the group head (GH). Pseudonym consumption is lower in this strategy. In [24], the author proposed a mechanism to preserve vehicles’ confidentiality throughout the journey to enhance the security of the VANET. When nodes come within the range of an RSU, it broadcasts a BSM. When neighbors receive this beacon, they send a BSM in return, including VID, pseudonym, location and speed. By using this information, the RSU confirms that vehicles are legal. Trip time informs when a vehicle departs from the current RSU. Afterward, trip time *T_i_* is calculated using Equation (1). *Range_RSU_* shows the transmission range of the RSU while *Speed_vehicle_* represents the vehicle’s speed. The vehicle’s speed is checked against the threshold speed *Vs*; if it is less than this, the vehicle enters into the congestion detection phase and transmits a congestion awareness beacon. For the confirmation of congestion, the RSU waits for other vehicles to send congestion messages. The advantage of this scheme is that unauthorized vehicles are reported and quick action is taken so that the adversary cannot listen to the communication of the vehicle. The drawback of this technique is that it is only suitable in heavy traffic.

(1) $$T_{i}=\frac{{Range}_{RSU}}{{Speed}_{vehicle}}$$

Yang et al. [25] presented a technique named the dynamic pseudonym swap zone (DPSZ), in which vehicles exchange their pseudonym by developing a temporary zone. In the case of any malicious activity, that vehicle’s credentials are revoked, and its exchanging procedure is also revoked. After it, the target vehicle is notified about it, and then allotted with a novel pseudonym. It will protect nodes from attacks planned by the adversary. The nodes can check their capability to respond according to Equation (2). *α* shows the likelihood of vehicles to reply to the initiator, |Þ*i*| represents the neighbors of vi, *µ* is the vehicle’s count to create a zone where vehicles can switch their pseudonyms and *e* is Euler’s constant. When |Þ*i;*| ≥ *µ*, in this case, nodes have little chance to response. This scheme is more secure against internal and external attacks. The weakness of this technique is that swapping occurs when vehicles reach a threshold *µ*. This perfect condition is not possible each time.

(2) $$\alpha =\left\{\begin{array}{c} 1,\left|Þi\right|=µ\\ e^{1\frac{\left|Þi\right|}{µ}},\left|Þi\right|\geq µ \end{array}\right.$$

During the silent mode, there is a great risk that vehicles are unable to receive safety beacons. In order to reduce this issue, vehicles update their pseudonym in the presence of *k* nodes. Furthermore, road traffic is dynamic and changes frequently; it enhances the anonymity set when more vehicles enter the silent mode. When the anonymity set increases, it ultimately increases adversary confusion. During time *t*, suppose *k* neighbors are available to change the pseudonym; then, at *t* = *t* + *at* time, vehicles have a choice to freely decide whether to change their pseudonym or not. If the beacon is transmitted with probability *p*, it represents vehicles that want to update their pseudonym; this procedure is called flickering. In *t* = *t* + *nT*, vehicles set the beacon bit to HT = 1 and inform new neighbors. So, that vehicle updates the pseudonym together at *t* = *t* + (*n* + 1) time. The duration of the silence mode decreases in comparison, to prevent bad effects on safety messages [26]. To prevent linking attacks and to increase privacy, another approach, the synchronized pseudonym-changing protocol (SPCP) [27], was proposed. In this scheme, vehicles change their pseudonym in the group that is monitored by a group head (GH). The advantage of this protocol is that it increases anonymization, and enhances the level of confusion for adversaries. The shortcoming of the scheme is that enormous storage is required for the TA so that the group record information can be handled easily.

### 2.2. Mix-Zone-Based Schemes

In mix-zone-based schemes, there are some zones (traffic signals, malls, marts, toll plazas) that are predefined. When vehicles enter these zones, they change their pseudonym. K.Emara et al. presented a scheme which allows vehicles to move into silent mode in case they enter the ideal region. When initiator vehicles find any silent node in their surroundings, they switch to silent mode too and then change their pseudonym [28]. This scheme proved to be better in the case of traceability. The drawback of the scheme is that the silent mode reduces safety-oriented applications. Li et al. [29] came up with a strategy to create a mix zone in the red traffic light. When vehicles stop at a red light, they become silent and change pseudonym. During a red light, not many essential beacons are neglected. Vehicles obtain active gain at green traffic lights. The scheme does not make a compromise on safety beacons during silent mode but is effective only with a high density. In [30], vehicles create a virtual cryptographic mix zone for changing pseudonym. In this zone, vehicles broadcast safety messages but in an encrypted format. After changing pseudonyms, vehicles exit from the zone. Safety messages are not neglected in this scheme but the decryption of beacons needs extra time. In [31], vehicles change pseudonyms in parking areas and shopping malls, and these places are considered as zones. Vehicles exit randomly from the zone, which increases the confusion of the adversary. In cases where zones are not available for a long time, vehicles will not change pseudonyms and the attacker can perform linking attacks on target vehicles easily.

In [32], one pseudonym is allotted per vehicle by the pseudonym certificate authority (PCA); after this, more pseudonyms are generated using a Gao algorithm. Pseudonym consumption is very low in this scheme but the randomization process is very challenging. In [33], when vehicles are in traffic, their speed is checked if it is slow (lies within 20 km/h to 40 km/h), and they check their neighbors. After ensuring the existence of neighbors, vehicles update their pseudonym. In order to encourage selfish nodes in the network to take part in the pseudonym-updating mechanism, a motivation procedure is used. Vehicles are given some incentive on changing pseudonym; if they will not change, their incentive value will be detected [34]. The benefit of the scheme is that it increases anonymity. The vehicular location privacy zone (VLPZ) is presented [35] in the network and it is divided into grids. Each grid contains zones where vehicles move and change pseudonyms. The entrance point is known as a router, and from which vehicles move into the zone and exit from the aggregator. The degree of anonymity is calculated using Equation (3), where *d* shows the degree of anonymity, *k* represents the capacity of the vehicular zone and |*AS*| shows the occupancy of the vehicular zone. This scheme needs a separate RSU, which is expensive to deploy.

(3) $$d=\frac{log2(|AS|)}{log2(k)}$$

In [36], vehicles opt for a group as per its velocity and change their pseudonym in cases where *S_th_* > 1, where *S_th_* represents the speed threshold. If a vehicle leaves a group to join another, it is also allowed to change pseudonym. The scheme is appropriate for long journeys but is not suitable for short distances.

## 3. System Model and Problem Statement

In this section, the system model of the proposed solution is described, which consists of four main entities which are the TA, vehicles, location-based server and RSU.

**Figure 2:**
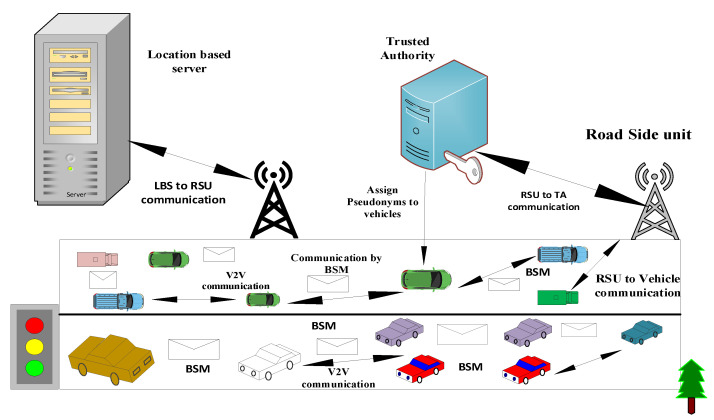
System models.

(1) The TA is used to allocate pseudonyms to vehicles when they enter the network. In case a vehicle is conducting suspicious activities in the network, after receiving the report from the RSU, the TA revokes the pseudonym of that vehicle. So, the main purpose of this entity is to allocate, revoke and keep the link between former and new pseudonyms.
(2) Vehicles are the basic components of the system model, which is equipped with the OBU, GPS and sensors. The vehicles can communicate with each other and the RSU for sharing safety beacons, and share pseudonym information and other information. During traveling on roads, vehicles need to know accurate information about their destination.
(3) The location-based server provides the following facilities: i) inquiring about vehicle appeal to the RSU, ii) sends a request to the location-based server (LBS) for providing accurate location information for moving to the desired destination.
(4) The RSU monitors traffic and informs vehicles about it in a timely manner. In this case, the pseudonyms are insufficient, and the RSU requests the TA to provide more numbers. In the case of malicious nodes in the network, the RSU instructs the TA to revoke its pseudonym. The system model of the proposed scheme is shown in Figure 2.

The core problem before broadcasting is that the vehicle’s actual distance is not considered, only the speed of the vehicle is noticed, and the BSM is transmitted. The topology in the IoVs is very dynamic: vehicles move at different speeds and follow different routes and lanes. So, there is a high chance that vehicles that are neighbors at time t will no longer remain neighbors at time Δ + t due to the large distance. However, they still receive a BSM [15]. This problem has a bad impact on pseudonym consumption. High pseudonym consumption increases the chances of an important BSM loss rate. When irrelevant vehicles receive a BSM, it disturbs a vehicle’s anonymity.

### Adversary Model

**Figure 3:**
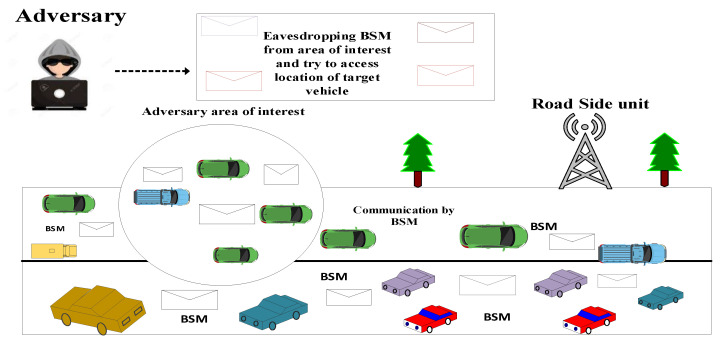
Adversary model.

An adversary is considered as somebody who spies on vehicles’ BSMs to obtain information about a vehicle’s location, direction and other sensitive information. The aim behind it is to threaten or trace drivers or passengers and follow the target vehicle’s path. After receiving a BSM, an adversary attempts to extract with the vehicle’s former pseudonym. With this aim, an adversary installs eavesdropping sensors into the trajectory to gain the BSMs. The adversary passively observes the BSMs from its area of interest but does not change the information available in the adversary model, as shown in Figure 3.

## 4. Efficient Pseudonym Consumption Protocol

We present the proposed efficient pseudonym consumption protocol (EPCP) that aims for the efficient utilization of a pseudonym. Vehicles may change their pseudonym when vehicle *v* has more neighbors. For sparse traffic, vehicles exchange their pseudonyms to avoid pseudonym wastage as well as increase anonymity. Besides the mix-context trend on which the EPCP scheme is based, there are some other methods that pseudonym-changing techniques have used. The silence-based pseudonym-changing trend refers to those cases that become silent for some specified or random time to change pseudonym. During the silent mode, vehicles do not broadcast or receive any safety messages. Fixed-place changing pseudonyms are those that change pseudonym only in front of a traffic red light signal, in parking lots near malls or markets, at road junctions, etc. The group-based changing pseudonym trend refers to those schemes that make groups on the basis of some metric and pseudonym-changing mechanisms that occur within groups. Many cases have used encryption-based pseudonym-changing trends that refer to mechanisms in which vehicles use encrypted beacons to transmit within their transmission range. The receiving vehicles first decrypt the information and then change pseudonym simultaneously, if needed.

The developed solution of the EPCP can be used for smooth and secure long and short journeys. It can be beneficial for military fleets, as the adversary cannot track all of the information all of the time, while such privacy issues exist in traditional transportation. Additionally, the scheme can be implemented for vehicles used for medical emergencies, and for lawyers that have security threats. The EPCP scheme can also be deployed for riding services and public transport, as the proposed scheme is not much more expensive to implement. On the whole, the EPCP is effective to use in all scenarios where anonymity is the main concern of users and passengers.

Before sending a BSM, vehicle *v* checks some metrics. In the first phase, vehicle *v* checks its neighbors as per the BSM received in the previous timeframe. After this, the next state is estimated. If the state lies within the premises of a close range then vehicles are considered to be relevant ones that are following the same state.

In the second phase, the speed of vehicles *v* is checked against two threshold values in contrast to the neighboring vehicles. If the relevant vehicles are moving too slow or too fast, this means that soon they will be far away from the premises of vehicle *v*. This results in increasing BSM delay. If speed is according to vehicle *v*, then its direction is checked as the vehicles can change route due to notifications received from the RSU.

In the third phase, if a vehicle’s *flagbit* is 1, then the pseudonym will be exchanged or changed as per the density of the road. In the case of sparse traffic when no vehicle lies in the close radius, then the pseudonym time is checked. After the expiry of the lifespan for the current pseudonym, the vehicle is allowed to change the pseudonym. To prevent a pseudonym-linking attack, we reduced the pseudonym lifespan in the proposed scheme. A list of notations used in this scheme is presented in Table 1.

The efficient pseudonym consumption algorithm is presented in Algorithm 1. In lines 1–7, when vehicle v obtains the BSM from its neighboring nodes, the position of the sending vehicles is extracted from the received BSM. If it lies within the transmission range, in this case, the BSM is kept; otherwise, it is discarded. The onboard unit of the vehicle helps it in interacting with nearby entities as well as sending and receiving BSMs. In the next time slot, vehicle v intends to send a BSM. After the beacon interval time, the BSM is prepared and important information about vehicle v is included in it. In lines 11–16, the BSM received in the previous time slot is checked, and if at least a single BSM of the vehicle is present, its next state is estimated. For the estimation of the next state, the *Kalman* filter is used. The difference between the present state and the estimated state is checked using Euclidean distance. If it lies in the close range then it is relevant and further parameters are checked.

In lines 20–29, the neighbor vehicle’s speed is checked against two speed values. In other schemes, only one threshold value is used, with the reason behind using two values being that vehicles that are too slow or too fast will quickly leave the proximity of vehicle v and will not remain its neighbor. If the road traffic is dense, then the vehicle will change its pseudonym; otherwise, it will be exchanged. In the case of no vehicle existing in proximity, then, after the pseudonym lifetime of the vehicle has expired, the vehicle changes its pseudonym. The pseudonym lifetime is decreased to 50 s to avoid a pseudonym-linking attack.
**Algorithm 1:** Efficient Pseudonym Consumption Algorithm//When intended vehicle v get BSM 1. N_position = BSM.pos (); 2. Neigh_dis = dis(my_position, N_position) 3. **If** (Neigh_dis ≤ T) **then** 4.   Neigh_v++ 5.   store ← store + Neigh_v; 6. **Else** drop BSM. 7. **End if**
   //intended vehicle v aims to disseminate BSM in upcoming timeslot 8. **while** (OBU status is active) **do** 9.     wait (beacon interval) 10.    Ready (BSM); 11. **if** (nodes ≥ k) **then** 12.    vehicles_trails ← kalman_filter(store); 13.     **for** i ← 1 to Neigh_v **do** 14.      **if** (Euclidean (vehicles_trails(i).pos, current_state.pos) ≤ Close_R) **then** 15.       adjacent ← adjacent + vehicles_trails(i); 16.      **End if** 17.     **End for** 18.     **if** (!adjacent.empty()) **then** 19.      Call Function Neighbor_speed ← BSM.speed() 20.      **if** (Neighbor_speed < threshold_min_) OR (Neighbor_speed > threshold_max_) **then** 21.       Call Function BSM (Delay) 22. **Else** 23.       N_direction = Call Function BSM_direction () 24.       **if** (std:: equal(mine_direction, N_direction)) **then** 25.         **if** (Neigh_v ≥ threshold &&((Neigh_v (Readyflag) && v_readyflag == 1)) **then** 26.          Call Function Update cooperatively pseudonym () 27.          Set Readyflag_bit to 0 28.         **elseif** (Neigh_v < threshold && ((Neigh_v (Readyflag) && v_readyflag == 1)) 29.            Random exchange of unused pseudonym (Vi, Vj) 30.            Set Readyflag_bit to 0 31.        **End if** 32.       **End if** 33.     **End if** 34. **If** (adjacent.empty()) **then** 35.     Locality ← False //no vehicle is in transmission range of vehicle v 36. **End if** 37. **If** (v_pseudolife > stable_span) **then** 38.    Call Function Update pseudonym (); 39.    Set Readyflag_bit to 0 40.  **End if** 41.**End if** 42.**End while**

## 5. Results and Discussion

In this section, we present the simulation environment, results and related discussions. To validate the results, we performed extensive simulations using privacy extension (PREXT) [37]. It is built upon the veins framework [38] which includes two main modules, which are Object Modular Network Testbed (OMNet++) version 5.0 [39] for network construction and Simulation of Urban Mobility (SUMO) 0.25.0 [40] for traffic mobility scenarios, as in the real world. The map of Munich city was used by downloading it from Open Street Map (OSM). For creating the vehicles’ route, randomTrips was employed. PREXT helped in analyzing crucial factors such as pseudonym consumption, traceability, normalized traceability and confusion rate, which are important factors from an anonymity perspective. For QoS, the BSM loss rate was checked. For simulation, a highway scenario was considered. The minimum and maximum speed thresholds were 5 m/s and 30 m/s, respectively. The base schemes were CPN [14], WHISPER [15] and DGVP [21]. A list of simulation parameters with respected values is shown in Table 2.

### 5.1. Average Percentage of Adversary Attains Traceability

**Figure 4:**
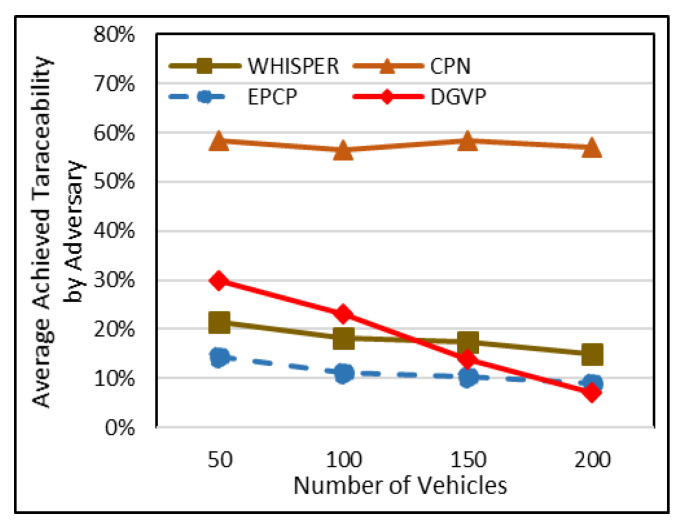
Average percentage of traceability in sparse to dense traffic scenario.

Traceability is a concept defined as the probability that an adversary will guess the target vehicle’s path appropriately using a BSM [28]. If the adversary knows the traces of the target vehicle, this increases its vulnerabilities and security threats. The higher the traceability, the lower the vehicle anonymization. So, it is a crucial parameter from an anonymization perspective; simulation was performed five times, and the average was considered under sparse to dense traffic. Figure 4 shows that the proposed scheme of the EPCP achieved the lowest traceability compared to the base schemes. The reason behind high traceability in CPN is that the techniques do not make use of sufficient triggers for changing pseudonyms. The lack of opting for a suitable context raises the chances of high traceability. WHISPER has relatively low traceability compared to CPN, which limits the transmission range on the basis of the speed of nearby vehicles. In the case of DGVP, initially, the traceability rate surges to 30%, but when the vehicles’ densities increase, the traceability factor starts dropping. The reason behind this is that this technique changes the pseudonym in groups. During sparse traffic, the few vehicles remain in the group and do not update the pseudonym until it has expired, whilst high-speed vehicles exit the group, making it easy for adversaries to trace vehicles. A crowd is formed as vehicle density increases, due to crowd vehicles changing to a slow speed and joining groups, changing the pseudonym together, which reduces the traceability factor. As can be observed, when the number of vehicles are 200, the traceability factor reduces to 7%. Our proposed EPCP checks multiple factors (direction, estimated next state of neighbors and direction) to minimize the chances of traceability. Besides this, in the EPCP, the pseudonym lifetime is also reduced to 50 s to lessen the possibility that an adversary creates a connection between a former and a new pseudonym correctly. In the case of sparse traffic on the road with 50 vehicles, CPN achieves 58.4% traceability, whereas WHISPER accomplishes 21.5% traceability, DGVP attains 30% and EPCP accomplishes 14.4% traceability.

### 5.2. Average Percentage of Adversary Attains Normalized Traceability

**Figure 5:**
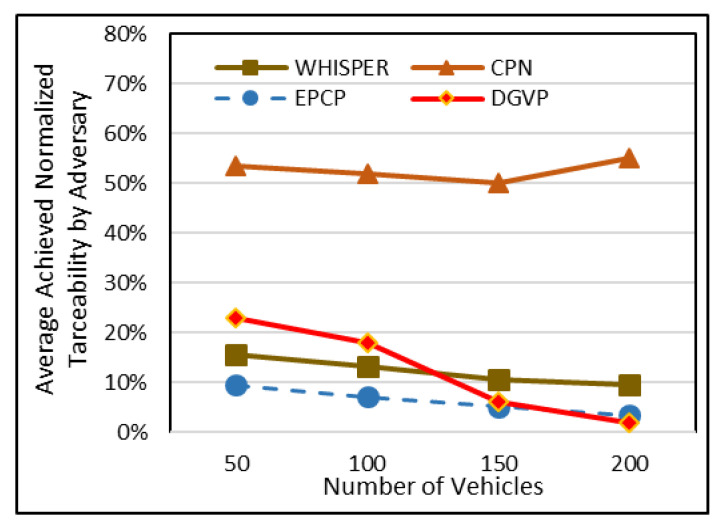
Average percentage of normalized traceability in sparse to dense traffic scenario.

Some vehicles do not update their pseudonym, and mapping out such vehicles is very easy. Eliminating such vehicles provides a better privacy level. This concept is known as normalized traceability [28]. Under this metric, a simulation can be conducted. Figure 5 depicts that the EPCP has significantly low normalized traceability. Under sparse traffic (when the number of vehicles are 50), after excluding those vehicles, the traceability ratio is reduced in CPN, and it attains normalized traceability of 54.4%. WHISPER lay within 16.5%, DGVP achieved normalized traceability of 23% and the proposed scheme of the EPCP had 9.5% normalized traceability. The results proved that EPCP and WHISPER had better normalized traceability in comparison to CPN.

### 5.3. Pseudonym Consumption

**Figure 6:**
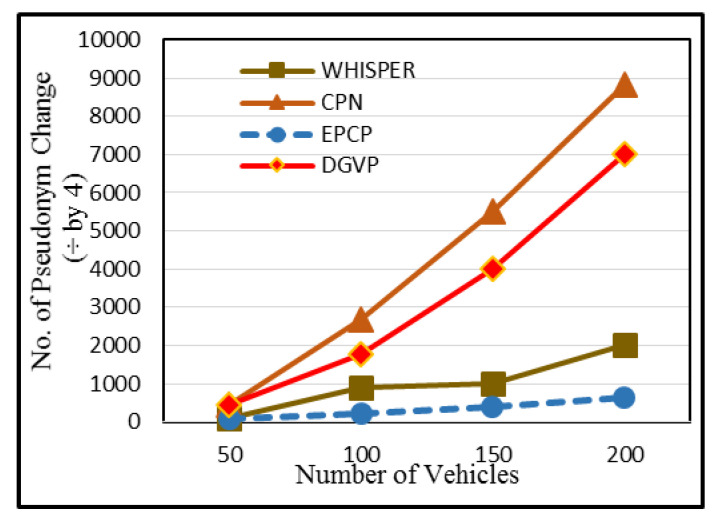
Pseudonym consumption.

Vehicles interact with other entities using a pseudonym. The TA provides a pair of private and public information to vehicles when they enter into a network for registration. For a pseudonym, the public key is considered. Vehicles have a sufficient set of pseudonyms; so, they must be used wisely. In the case of low pseudonyms, vehicles appeal to the RSU to request the TA to allot them more pseudonyms. In return, the TA provides vehicles more pseudonyms through the RSU. This increases communication and computation overhead and makes the scheme costly to deploy. In CPN, pseudonym utilization is very high; the reason behind this is that when a vehicle wants to update its pseudonym, all neighboring nodes in the network also update their pseudonym even without any need, which ultimately raises pseudonym consumption. Moreover, vehicles also update their pseudonym when they meet a trigger (a trigger is a condition when *k* number of neighbors are present), and the value of *k* is kept as 2 within it. Although WHISPER has lower pseudonym consumption than CPN, it should be even less. The WHISPER scheme only uses the metric of speed before sending a BSM, and many neighbor vehicles can change their lanes after some time, but they still change their pseudonyms without any need. In DGVP, vehicles make use of two pseudonyms: one is original, and one is virtual. During the virtual method, two messages are generated with pseudonyms and are transmitted to member vehicles. This mechanism increases pseudonym utilization. The proposed scheme has lower pseudonym consumption, as shown in Figure 6, because only those vehicles that will remain for some time change pseudonyms. If such vehicles do not exist in the network, the BSM is delayed for some time to avoid the wastage of pseudonyms. During a dispersed distribution of vehicles on the road with 50 vehicles, the pseudonym utilization is 440 in CPN. For WHISPER, the pseudonym consumption is 103, in DGVP it lies in the range of 430 and in EPCP it remains at 50.

### 5.4. BSM Loss Rate

**Figure 7:**
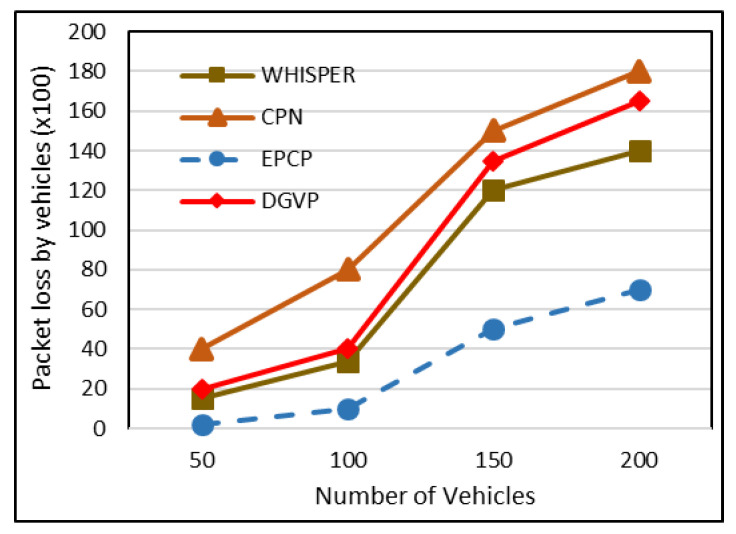
BSM packet loss rate.

Vehicles possess a limited buffer to store the beacons received from various entities. The vehicles receive irrelevant BSMs and may keep them for a long time. This results in filling the buffers, which causes emergency messages to be delayed or dropped. The existing techniques retain the BSMs of these vehicles in buffer that takes different paths at time Δ + t which are not useful now; this rises the BSM loss rate. In the proposed technique of the EPCP, when vehicles receive BSMs outside of the close area, it drops them, which lowers the BSM loss rate. Besides this, the proposed scheme generates and transmits BSMs at a very stable rate, which helps in overcoming the loss rate, while in the CPN and WHISPER strategies, both keep irrelevant BSMs, which increases the chances of important BSMs being lost. In DGVP, vehicles share information about safety only to their group members. So, when few vehicles lie within a group, the BSM loss rate is low, while it increases with increasing increments of group members. The BSM packet loss is stable in the EPCP, compared to WHISPER, DGVP and CPN, as presented in Figure 7. The value on the *X*-axis indicates the total number of vehicles, whereas values on the *Y*-axis represent the BSM loss rate. The loss rate in WHISPER lies within the range of 1500, 3400, 12,000 and 14,000, and the numbers of vehicles are 50, 100, 150 and 200, correspondingly. Similarly, the BSM loss in CPN is up to 4000, 8000, 15,000 and 18,000, with 50, 100, 150 and 200 vehicles. In DGVP, it remains at 2000, 4000, 13,500 and 16,500 under vehicle densities of 50, 100, 150 and 200. In the proposed scheme of the EPCP, the loss rate is up to 200, 1000, 5000 and 7000 under vehicle densities of 50, 100, 150 and 200. The result signifies that the EPCP has a lower loss rate than CPN and WHISPER.

### 5.5. Average Confusion for Attacker Due to Change in Pseudonym

**Figure 8:**
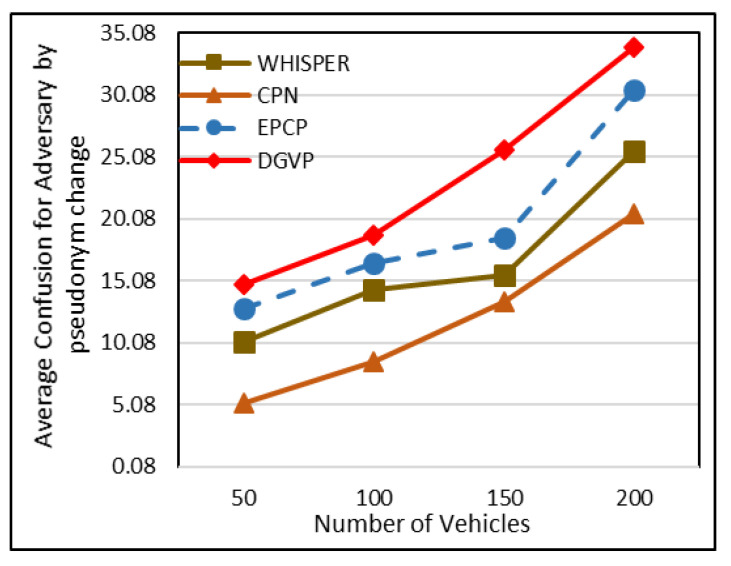
Average confusion for adversary according to pseudonym change.

By creating high confusion for an adversary, better anonymity can be achieved that ultimately increases the privacy level. Different vehicle densities (sparse, mediocre and dense) are shown in the *X*-axis, while the *Y*-axis shows the average confusion level for the adversary (the results are shown in Figure 8). The higher confusion rate in the EPCP is because direction and speed threshold factors are considered before sending a pseudonym-changing beacon. It adds the minimum and only relevant vehicles that overcome the possibility of attacks. Apart from this, in sparse situations, pseudonyms are exchanged randomly with each other so that pseudonyms should not be wasted and upsurge the confusion of attackers in tracing the target vehicle. In DGVP, during dense traffic, vehicles slow down their speed which increases the anonymity set, which increases confusion for the adversary in mapping out the target vehicle accurately in the case of the disperse distribution of traffic when the number of vehicles are 50. WHISPER accomplishes a value of 10.2, whereas the proposed scheme of the EPCP maintains an average value of 12.8, DGVP accomplishes an average value of 10.8 and CPN attains an average value of 5.2. During high traffic, the average confusion rate is up to 25.5, 30.5, 33.9 and 20.5 for WHISPER, EPCP, DGVP and CPN, respectively.

### 5.6. Proportion of Vehicles That Changed Pseudonym

When a stable proportion of vehicles updates the pseudonym cooperatively, it surges the efficiency of the technique, while updating the pseudonym very frequently upsurges the communication and computation cost. In the context of the CPN, it had a very high proportion of vehicles that changed pseudonyms because of a trigger (a condition when two vehicles exist in the transmission range), and it changed pseudonyms.

**Figure 9:**
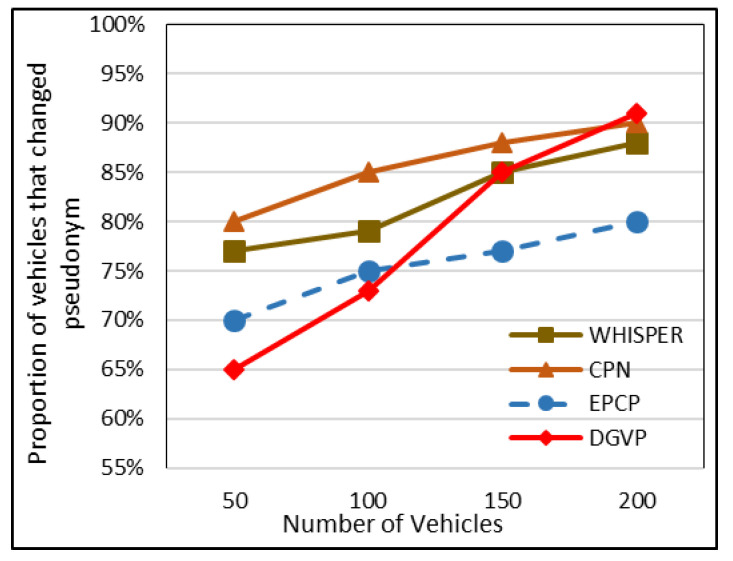
Proportion of vehicles that changed their pseudonym.

The EPCP had a slightly low proportion of vehicles that changed pseudonyms; because of strict checks, some vehicles showed a lack of interest in changing their pseudonym. WHISPER had a worthy proportion of vehicles that cooperatively updated their pseudonyms. As far as the DGVP is concerned, initially it had a lower vehicle proportion of those changing pseudonyms, but when the density of traffic became heavy, the proportion of vehicles that changed their pseudonym significantly increased. Figure 9 shows that in the EPCP, the proportion of vehicles that changed their pseudonym remained at 70%, 75%, 77% and 80% under traffic of 50, 100, 150 and 200 vehicles, respectively. The WHISPER proportion lay under 77–89% in sparse and dense traffic. The CPN lay within the proportion of 82% in the case of sparse traffic, while this proportion increased up to 90% in dense traffic. In DGVP, the proportion remained at 65%, 71%, 85% and 91% with traffic of 50, 100, 150 and 200 vehicles, correspondingly.

Overall, the performance of the proposed scheme remained stable under various metrics from sparse to dense traffic, but the shortcoming of the EPCP scheme is that slightly lower vehicles changed their pseudonym because of selfish nodes in the network. The WHISPER scheme performed fairly for most of the metrics. In the case of the CPN scheme, the pseudonyms were not well utilized, which ultimately increased the computation and communication overheads. As DGVP is a dense-based scheme, it outperforms in dense traffic, while the effectiveness is reduced in distributed traffic. So, DGVP is only acceptable to use in heavy traffic.

## 6. Conclusions

In this paper, a mix-context technique named the efficient pseudonym consumption protocol was proposed to reduce pseudonym utilization by sending beacons when relevant neighboring vehicles were present on the road. For this purpose, the next state of vehicles, their direction and their speed threshold were checked. In the proposed strategy, vehicles are allowed to exchange pseudonyms in lower traffic and change only when traffic is dense to utilize pseudonyms effectively. Simulation was performed to check the effectiveness of the proposed scheme of the EPCP under the PREXT simulator, along with OMNet++ and SUMO. The results showed that the proposed technique of the EPCP has better pseudonym consumption, a low BSM loss rate and a higher confusion rate for adversaries, and achieved low traceability and normalized traceability compared to the existing schemes of CPN, WHISPER and DGVP when traffic was sparse. The limitation of the scheme is that no motivation mechanism is introduced to encourage selfish nodes to participate in the pseudonym-changing process. For the proposed work, only external adversary was considered, which may not be very efficient for cases of internal adversary. In the near future, an encouragement-based mechanism will be introduced to motivate selfish nodes in the network to participate in the pseudonym-changing process to increase the proportion of vehicles. Besides this, a scenario of an internal adversary should also be checked when some internal entities, i.e., the vehicle or RSU, become semi-honest or malicious. Additionally, the communication cost of the proposed scheme should also be checked, and the EPCP should be compared with other anonymity-based schemes; these are a few of our upcoming plans.

## Figures and Tables

**Table 1:** List of notations.

Sr.	Notation	Description
1.	k	Number of neighbors
2.	Neigh_dis	Neighbor distance
3.	Neigh_v	Vehicles in locality of vehicle v
4.	threshold_min_	Minimum threshold speed
5.	threshold	Neighbor threshold value
6.	Vi	Vehicle v
7.	Vj	Neighboring vehicles
8.	thresholdmax	Maximum threshold speed
9.	Close_R	Close range
10.	N_direction	Direction of neighbor vehicles

**Table 2:** Simulation parameters and values.

Parameters	Values
Simulation time	300 s
Number of vehicles	50, 100, 150, 200
Transmission range	300 m
Pseudonym stable time	50 s
Minimum speed threshold	5 m/s
Maximum speed threshold	30 m/s
Close range	100 m
Neighbor threshold	40
Operating system	Ubuntu 16.04
Coupling protocol	TraCi

## Data Availability

Will be available on request.

## References

[1] Fadhil J.A., Sarhan Q.I. Internet of Vehicles (IoV): A Survey of Challenges and Solutions. Proceedings of the 2020 21st International Arab Conference on Information Technology (ACIT).

[2] Sharma S., Kaushik B. (2019). A survey on internet of vehicles: Applications, security issues & solutions. Veh. Commun..

[3] Hasan M., Mohan S., Shimizu T., Lu H. (2020). Securing vehicle-to-everything (V2X) communication platforms. IEEE Trans. Intell. Veh..

[4] Karagiannis G., Altintas O., Ekici E., Heijenk G., Jarupan B., Lin K., Weil T. (2011). Vehicular networking: A survey and tutorial on requirements, architectures, challenges, standards and solutions. IEEE Commun. Surv. Tutor..

[5] Ji B., Zhang X., Mumtaz S., Han C., Li C., Wen H., Wang D. (2020). Survey on the Internet of Vehicles: Network Architectures and Applications. IEEE Commun. Stand. Mag..

[6] Khan M.Z., Alhazmi O.H., Javed M.A., Ghandorh H., Aloufi K.S. (2021). Reliable internet of things: Challenges and future trends. Electronics.

[7] Lin K., Li C., Li Y., Savaglio C., Fortino G. (2020). Distributed learning for vehicle routing decision in software defined Internet of vehicles. IEEE Trans. Intell. Transp. Syst..

[8] Talat H., Nomani T., Mohsin M., Sattar S. A Survey on Location Privacy Techniques Deployed in Vehicular Networks. Proceedings of the 2019 16th International Bhurban Conference on Applied Sciences and Technology (IBCAST).

[9] Garg T., Kagalwalla N., Churi P., Pawar A., Deshmukh S. (2020). A survey on security and privacy issues in IoV. Int. J. Electr. Comput. Eng..

[10] Wang J., Shao Y., Ge Y., Yu R. (2019). A survey of vehicle to everything (V2X) testing. Sensors.

[11] Talib M.S., Hassan A., Hussin B., Hassan A.A.H. (2018). Vehicular Ad-hoc networks: Current challenges and future direction of research. J. Adv. Res. Dyn. Control Syst..

[12] Ferrag M.A., Maglaras L., Ahmim A. (2017). Privacy-Preserving Schemes for Ad Hoc Social Networks: A Survey. IEEE Commun. Surv. Tutor..

[13] Afzal Z., Kumar M. (2020). Security of Vehicular Ad-Hoc Networks (VANET): A survey. J. Phys. Conf. Ser..

[14] Pan Y., Li J. (2013). Cooperative pseudonym change scheme based on the number of neighbors in VANETs. J. Netw. Comput. Appl..

[15] Babaghayou M., Labraoui N., Ari A.A.A., Ferrag M.A., Maglaras L., Janicke H. (2021). Whisper: A location privacy-preserving scheme using transmission range changing for internet of vehicles. Sensors.

[16] Li X., Zhang H., Ren Y., Ma S., Luo B., Weng J., Ma J., Huang X. (2020). PAPU: Pseudonym Swap with Provable Unlinkability Based on Differential Privacy in VANETs. IEEE Internet Things J..

[17] Zidani F., Semchedine F., Ayaida M. (2018). Estimation of Neighbors Position privacy scheme with an Adaptive Beaconing approach for location privacy in VANETs. Comput. Electr. Eng..

[18] Singh P.K., Gowtham S.N., Tamilselvan S., Nandi S. (2019). CPESP: Cooperative Pseudonym Exchange and Scheme Permutation to preserve location privacy in VANETs. Veh. Commun..

[19] Bouksani W., Bensaber B.A. (2019). RIN: A dynamic pseudonym change system for privacy in VANET. Concurr. Comput. Pract. Exp..

[20] Emara K., Woerndl W., Schlichter J. (2016). Context-based Pseudonym Changing Scheme for Vehicular Adhoc Networks. arXiv.

[21] Ullah I., Shah M.A., Khan A., Maple C., Waheed A. (2021). Virtual pseudonym-changing and dynamic grouping policy for privacy preservation in vanets. Sensors.

[22] Ullah I., Shah M.A., Khan A., Maple C., Waheed A., Jeon G. (2021). A distributed mix-context-based method for location privacy in road networks. Sustainability.

[23] Ullah I., Shah M.A., Khan A. Adaptive Grouping and Pseudonym Changing Policy for Protection of Vehicles Location Information in VANETs. Proceedings of the 2021 IEEE Symposium Series on Computational Intelligence (SSCI).

[24] Wahid A., Yasmeen H., Shah M.A., Alam M., Shah S.C. (2019). Holistic approach for coupling privacy with safety in VANETs. Comput. Netw..

[25] Yang M., Feng Y., Fu X., Qian Q. (2019). Location privacy preserving scheme based on dynamic pseudonym swap zone for Internet of Vehicles. Int. J. Distrib. Sens. Netw..

[26] Zhang Z., Feng T., Sikdar B., Wong W.C. A Flickering Context-based Mix Strategy for Privacy Protection in VANETs. Proceedings of the IEEE International Conference on Communications.

[27] Weerasinghe H., Fu H., Leng S., Zhu Y. Enhancing unlinkability in vehicular ad hoc networks. Proceedings of the 2011 IEEE International Conference on Intelligence and Security Informatics.

[28] Emara K., Woerndl W., Schlichter J. CAPS: Context-aware privacy scheme for VANET safety applications. Proceedings of the 8th ACM Conference on Security & Privacy in Wireless and Mobile Networks, WiSec.

[29] Li Y., Yin Y., Chen X., Wan J., Jia G., Sha K. (2021). A Secure Dynamic Mix Zone Pseudonym Changing Scheme Based on Traffic Context Prediction. IEEE Trans. Intell. Transp. Syst..

[30] Ying B., Makrakis D., Mouftah H.T. (2013). Dynamic mix-zone for location privacy in vehicular networks. IEEE Commun. Lett..

[31] Lu R., Lin X., Luan T.H., Liang X., Shen X. (2012). Pseudonym changing at social spots: An effective strategy for location privacy in VANETs. IEEE Trans. Veh. Technol..

[32] Xu X., Huang Q., Zhu H., Sharma S., Zhang X., Qi L., Bhuiyan M.Z.A. (2020). Secure service offloading for internet of vehicles inSDN-enabled mobile edge computing. IEEE Trans. Intell. Transp. Syst..

[33] Shaleesh I., Almohammedi A., Mohammad N., Ahmad A., Shepelev V. (2021). Cooperation and radio silence strategy in Mix Zone to Protect Location Privacy of Vehicle in VANET. Tikrit J. Eng. Sci..

[34] Ying B., Makrakis D. (2015). Reputation-based Pseudonym Change for Location Privacy in vehicular networks. IEEE Int. Conf. Commun..

[35] Boualouache A., Senouci S.M., Moussaoui S. Towards an efficient pseudonym management and changing scheme for vehicular ad-hoc networks. Proceedings of the 2016 IEEE Global Communications Conference (GLOBECOM).

[36] Ullah I., Wahid A., Shah M.A., Waheed A. VBPC: Velocity Based Pseudonym Changing Strategy to Protect Location Privacy of Vehicles in VANET. Proceedings of the 2017 International Conference on Communication Technologies (ComTech).

[37] Emara K. Poster: PREXT: Privacy extension for Veins VANET simulator. Proceedings of the IEEE Vehicular Networking Conference (VNC).

[38] Sommer C., Eckhoff D., Brummer A., Buse D.S., Hagenauer F., Joerer S., Segata M. (2019). Veins: The Open Source Vehicular Network Simulation Framework.

[39] Varga A., Hornig R. An overview of the OMNeT++ simulation environment. Proceedings of the SIMUTools 2008—1st International ICST Conference on Simulation Tools and Techniques for Communications, Networks and Systems.

[40] Krajzewicz D., Erdmann J., Behrisch M., Bieker L. (2012). Recent Development and Applications of SUMO—Simulation of Urban MObility. Int. J. Adv. Syst. Meas..

